# Multiple forms of discrimination and internalized stigma compromise retention in HIV care among adolescents: findings from a South African cohort

**DOI:** 10.1002/jia2.25488

**Published:** 2020-05-21

**Authors:** Marija Pantelic, Marisa Casale, Lucie Cluver, Elona Toska, Mosa Moshabela

**Affiliations:** ^1^ University of Sussex Brighton and Sussex Medical School Brighton UK; ^2^ University of Oxford Department of Social Policy and Intervention Oxford UK; ^3^ University of the Western Cape School of Public Health Cape Town South Africa; ^4^ University of Cape Town Department of Psychiatry and Mental Health Cape Town South Africa; ^5^ University of Cape Town Department of Sociology Cape Town South Africa; ^6^ University of Cape Town Centre for Social Science Research Cape Town South Africa; ^7^ University of KwaZulu‐Natal Howard College School of Nursing and Public Health Durban South Africa

**Keywords:** HIV, stigma, discrimination, adolescent, retention, adherence

## Abstract

**Introduction:**

Efficacious antiretroviral treatment (ART) enables people to live long and healthy lives with HIV but young people are dying from AIDS‐related causes more than ever before. Qualitative evidence suggest that various forms of HIV‐related discrimination and resulting shame act as profound barriers to young people’s engagement with HIV services. However, the impact of these risks on adolescent retention in HIV care has not been quantified. This study has two aims: (1) to examine whether and how different types of discrimination compromise retention in care among adolescents living with HIV in South Africa; and (2) to test whether internalized stigma mediates these relationships.

**Methods:**

Between 2014 and 2017, adolescents living with HIV (aged 10 to 19) from 53 health facilities in the Eastern Cape, South Africa, were interviewed at baseline (n = 1059) and 18‐month follow‐up (n = 979, 92.4%), with responses linked to medical records. Data were analysed through multiple regression and mediation models.

**Results:**

About 37.9% of adolescents reported full retention in care over the 2‐year period, which was associated with reduced odds of viral failure (OR: 0.371; 95% CI: .224, .614). At baseline, 6.9% of adolescents reported discrimination due to their HIV status; 14.9% reported discrimination due to HIV in their families and 19.1% reported discrimination in healthcare settings. Healthcare discrimination was associated with reduced retention in care both directly (effect: −0.120; CI: −0.190, −0.049) and indirectly through heightened internalized stigma (effect: 0.329; 95% CI: 0.129, 0.531). Discrimination due to family HIV was associated with reduced retention in care both directly (effect: −0.074, CI: −0.146, −0.002) and indirectly through heightened internalized stigma (effect: 0.816, CI: 0.494, 1.140). Discrimination due to adolescent HIV was associated with reduced retention in care only indirectly, through increased internalized stigma (effect: 0.408; CI: 0.102, 0.715).

**Conclusions:**

Less than half of adolescents reported 2‐year retention in HIV care. Multiple forms of discrimination and the resultant internalized stigma contributed to this problem. More intervention research is urgently needed to design and test adolescent‐centred interventions so that young people living with HIV can live long and healthy lives in the era of efficacious anti‐retroviral treatment.

## 1 INTRODUCTION

There are 1.8 million adolescents living with HIV globally, of whom 1.5 million (85%) are in Sub‐Saharan Africa [1]. At a time when efficacious antiretroviral treatment (ART) enables people living with HIV to live long and healthy lives, young people between the ages of 13 and 24 are the only age group for whom AIDS‐related mortality is on the rise [2]. The exceptionally low rates of ART adherence and retention in HIV care in this age group [3, 4] have contributed to AIDS being the leading cause of death among adolescents in Africa [1]. But there is limited evidence of successful interventions to support adolescent retention in HIV care and their long‐term survival. Existing intervention research is focused on clinic‐based interventions [5], which are essential but insufficient to meet the complex needs of adolescents living with HIV [3, 4], including adolescents who are not engaging with the health system.

Adolescents living with HIV experience multiple forms of stigma. Stigma is commonly defined as a process through which individuals are “disqualified from full social acceptance” due to an undesirable “label” [6], such as an HIV diagnosis. According to Goffman, the experience of stigma reduces people from being seen as “whole” and “usual” to being seen as “tainted” and ‘discounted’ [6]. According to the HIV stigma framework, stigma can be broadly categorized into discrimination and internalized stigma [7]. Discrimination refers to overt instances of being treated differently from others due to a person’s HIV status [7]. Adolescents living with HIV are subjected to discrimination within their communities and families both because of their own HIV status and, sometimes, because of their caregivers’ HIV status [8, 9, 10]. Adolescents living with HIV also report frequent instances of healthcare providers getting angry and shouting at them, which they experience as discrimination [3]. Being a target of discrimination can lead to internalized stigma [11], which occurs when adolescents internalize stigmatizing characterizations of people living HIV and accept them as applicable to themselves [12]. Internalized stigma evokes feelings of shame, guilt, helplessness and, in some cases, suicidal ideation [10, 13, 14, 15, 16]. A national survey of more than 10,000 people living with HIV in South Africa found that 36% reported experiencing discrimination and 43% reported experiencing internalized HIV stigma in the past year. Young people living with HIV (ages 15 to 24) had a higher risk of both discrimination victimization and internalized stigma than any older age group [17].

Qualitative evidence on adolescent HIV and the broader literature on adolescent development suggests that HIV‐related discrimination and internalized shame act as profound barriers to engagement with health services among young people living with HIV [10, 15, 16, 18, 19]. Adolescence is a time when self‐conceptions develop [20]. Difficult physical, psychosocial and intellectual transitions are known to compromise the self‐worth and general self‐esteem of adolescents [21]; but adolescents living with HIV experience added complexities, which can further compromise their self‐image [22]. Adolescents living with HIV often experience physical and/or cognitive difficulties [23, 24], which can make them “stand out” from their peers. For example cognitive difficulties often result in poor educational outcomes for young people living with HIV relative to their peers [25]. The lower weight and height‐for‐age experienced by adolescents born with HIV [23] is recognized by young people in high HIV‐prevalence settings to be a marker of HIV [26]. These difficulties make adolescents living with HIV particularly vulnerable to discrimination and the ensuing internalized‐stigma [10].

The effects of discrimination on adolescent long‐term retention in HIV care have not yet been quantified [27]. The link between HIV‐related discrimination and internalized stigma has been quantitatively tested in both adolescent [10] and adult populations [11]. A growing body of adult‐focused evidence suggests that internalized stigma has onward effects on retention in HIV care [15, 28, 29], suggesting a potential pathway from discrimination to compromised retention in care via internalized stigma. However, this has not been empirically tested among adolescents living with HIV. Moreover, the divergent forms of discrimination experienced by adolescents living with HIV have been understudied. To our knowledge, adolescent‐focused quantitative studies have examined adolescent experience of discrimination due to their own HIV status [10] or their caregivers’ status [8] but never both and never with the added dimension of discrimination in healthcare settings.

This study has two aims: (1) to examine whether different types of discrimination are associated with retention in care among adolescents living with HIV and (2) to test whether internalized stigma mediates this relationship. We report on data from a total population study of adolescents living with HIV (n = 979) in a high‐prevalence health district in South Africa.

## 2 METHODS

The study was developed in collaboration with the South African Departments of Health and Basic Education, UNICEF, PEPFAR‐USAID, regional and local NGOs and an advisory group of 20 South African adolescents with the primary aim of understanding non‐adherence and non‐retention in HIV care among adolescents living with HIV. The adolescent advisory group was involved in developing the questionnaire and study procedures, and remained engaged throughout the study through regular camps and knowledge exchange workshops.

Adolescents living with HIV (aged 10 to 19 at baseline) in a high HIV‐prevalence resource‐limited health district in the Eastern Cape, South Africa were interviewed at baseline (n = 1059) and 18‐month follow‐up (n = 979), with their responses linked to clinical data were available (n = 514, 52.5%). Baseline data collection took place over the course of 2014 to 2015, and follow‐up was in 2016 to 2017.

All adolescents who had ever initiated HIV treatment in 53 government‐run health facilities were identified through clinic patient records and traced back to 180 communities, to ensure inclusion of those not engaged in care [3]. Adolescents were interviewed in their first language (97% Xhosa) and could choose their place of interview (either at the clinics or in their homes, schools or other locations they identified as safe). Questionnaires were tablet‐based, allowing adolescents to answer sensitive questions privately, without showing their answers to the research assistant. Research assistants were available to help in cases of low literacy.

Ethical clearance was provided by Oxford University (SSD/CUREC2/12‐21), the University of Cape Town (CSSR 2013/4), the South African National Departments of Health, Basic Education and Social Development and the Eastern Cape Departments of Health, Basic Education and Social Development. All adolescents and their primary caregivers gave written informed consent for participation. No financial incentives were given, but all adolescents received a snack, small gift pack including sanitary products and stationary (selected by the project’s adolescent advisory group) and a certificate with information about support services, regardless of whether they consented to participate or completed their interviews. Confidentiality was maintained except in cases of risk of harm: a total of 94 referrals were made to health or social services when participants reported abuse, suicidal attempts, rape or severe untreated illness.

### 2.1 Measures

*Outcome measure: Retention in care* was defined as both no missed clinic visits in the past year and> 85% ART adherence over the past week [3], reported by the adolescent at both baseline and follow‐up. Adolescent self‐report was used due to low rates of recording of appointments in patient files and high rates of adolescent mobility between clinics. ART adherence was measured over the past week in order to minimize recall bias, via an adapted version of the Patient Medication Adherence Questionnaire [30, 31]. Viral loads were extracted from clinical records at follow up to compute virologic failure (defined as a viral load of more than 1000 copies/ml) [32] and validate the adolescent‐reported outcome measure.

#### 2.1.1 Baseline measures

*Discrimination:* Three different types of discrimination were measured, all using adolescent self‐report for the past year: 1 – *discrimination due to the adolescent’s HIV status* measured via discrimination items from the validated adolescents living with HIV stigma scale, which included items on being teased or losing friends due to HIV [16]; 2 – *discrimination due to a family member’s HIV status* measured via the standardized stigma‐by‐association scale, which included items about having been gossiped about or treated badly due to a family member’s HIV status [33] and 3 – *discrimination in healthcare settings* measured through a question about frequency of being shouted at by a healthcare provider [3]. A ‘*discrimination multiplicity’* variable was additionally computed to denote the number of different types of discrimination experienced. A variable with three categories was computed, with values 0, 1 and 2 denoting no discrimination, 1 type of discrimination and multiple types of discrimination respectively.

*Internalized stigma* was measured through five items from the adolescents living with HIV stigma scale, including items on feeling ashamed or different from other young people due to HIV [16]. Response options ranging from 0 (never) to 2 (most of the time) were summed into a scale variable. The Chronbach alpha reliability statistic for the scale in this sample was 0.736.

#### 2.1.2 Baseline socio‐demographic and HIV‐related covariates

Six baseline covariates were controlled for in all analyses, using adolescent self‐report and clinical records: [1] age (dichotomized as younger adolescents aged 10 to 14 and older adolescents aged 15 to 19); (2) sex; (3) residential location (urban/rural); (4) vertical/horizontal HIV transmission was assessed following a literature‐informed algorithm used for existing Sub‐Saharan African paediatric HIV cohorts [4]; (5) length of time on ART was measured via self‐report and clinic records (more/less than one year on treatment) and (6) adolescents’ knowledge of their own HIV status. All covariates were dichotomous.

### 2.2 Analysis strategy

**Figure 1:**
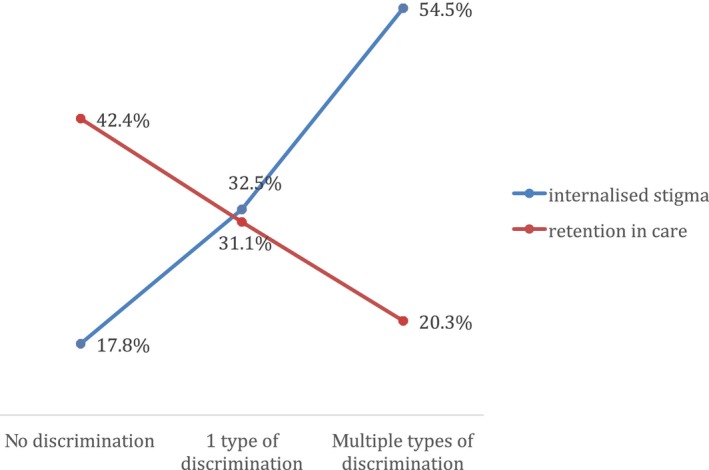
Frequencies of internalized stigma and retention in care by discrimination multiplicity.

**Figure 2:**
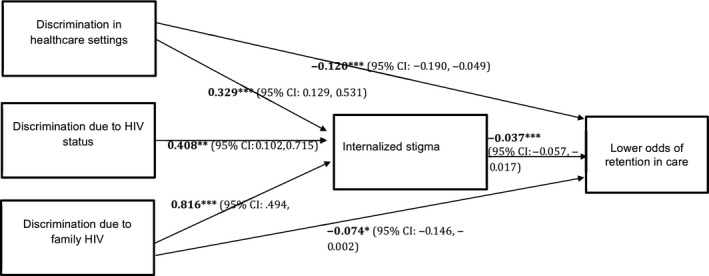
Mediation model results.

Analyses were conducted in seven stages using SPSS 23 and STATA 14. First, frequency distributions for all outcomes, hypothesized predictors and covariates were reported. Second, cross‐tabulations were run to describe associations between discrimination multiplicity against internalized stigma and retention in care (Figure 1). Third, eligible participants included in the study were compared to those excluded (the 9.9% not traceable or refused participation) on the sociodemographic characteristics that were available for both groups (age, gender, urban/rural location) using chi‐square tests. Fourth, baseline characteristics of adolescents who were re‐interviewed were compared to those who were not re‐interviewed at follow‐up. Fifth, the self‐reported retention in care outcome variable was validated against virological failure, controlling for potential covariates in a multivariate logistic regression (Table 1).Sixth, we examined the relationship between multiple forms of discrimination and retention in care. A multiple logistic regression was run with the three baseline discrimination variables, baseline internalized stigma and baseline covariates as independent variables and retention in care (at both baseline and follow up) as the dependent variable. Lastly, a mediation model was run with the three discrimination variables as independent variables, internalized stigma as the mediator and retention in care as the dependent variable, controlling for covariates (Figure 2).

**Table 1:** Descriptive statistics for final sample of adolescents (N = 979)

Variables	N (% sample)
Socio‐demographic characteristics	
Age	
Above 15	362 (37.0%)
Below 15	617 (63.0%)
Gender	
Female	539 (55.1%)
Male	440 (44.9%)
Location	
Rural	261 (26.7%)
Urban	716 (73.1%)
Missing	2 (0.2%)
HIV‐ and treatment‐related characteristics	
Mode of HIV transmission	
Horizontal	205 (20.9%)
Vertical	774 (79.1%)
Time on Treatment	
More than a year	808 (82.5%)
Less than a year	171 (17.5%)
Knowledge of status	
Knows status	737 (75.3%)
Does not know status	230 (23.5%)
Missing	12 (1.2%)
Retention in care	
Yes	371 (37.9%)
No	608 (62.1%)
Experiences of discrimination and internalized stigma	
Discrimination due to HIV status	
Yes	68 (6.9%)
No	911 (93.1%)
Discrimination due to a family member’s HIV status	
Yes	146 (14.9%)
No	833 (85.1%)
Discrimination in healthcare settings	
Yes	187 (19.1%)
No	792 (80.9%)
Discrimination multiplicity	
No discrimination	654 (66.8%)
1 type of discrimination	256 (26.1%)
>1 type of discrimination	69 (7.0%)
Internalized stigma	
Yes	229 (23.4%)
No	720 (73.5%)
Missing	30 (3.0%)

## 3 RESULTS

### 3.1 Sample characteristics

Descriptive characteristics of the sample are provided in Table 1. At baseline, the majority of adolescents in the sample were below 15 years of age (63%); female (55.1%); residing in urban areas (73.1%); had contracted HIV vertically (79.1%); were aware of their HIV status (75.3%); and had been on ART for over a year (82.5%).

About 37.9% of adolescents reported retention in care over the two‐year period, and 33.1% of participants reported having experienced HIV‐related discrimination in the past year. Of these, 6.9% reported having experienced discrimination due to their HIV status; 14.9% reported having experienced discrimination because of a family member’s HIV status and 19.1% reported having experienced discrimination by their healthcare providers.

#### 3.1.1 Reliability of sample and outcome measure

At baseline, 90% (n = 1059) of eligible adolescents identified through clinic records (n = 1176) were interviewed. Of those that were not reached, 4% refused participation (either adolescent or caregiver refusal); 1% was unable to participate because of severe cognitive delay and 5% were not traceable. No significant differences on age, gender or rurality were found between the interviewed and non‐interviewed adolescents [3]. Of the adolescents interviewed at baseline, 92.4% (n = 979) were re‐interviewed at follow up. Of the approximately 7.6% of adolescents who were not re‐interviewed at follow‐up, approximately 1% had passed away; 1.7% refused participation and 4.9% could not be traced. Adolescents who were re‐interviewed at follow‐up were on average 10 months younger and lived in rural areas, with higher rates of maternal orphanhood [34].

### 3.2 Associations between retention in care and viral load clinical data

Retention in care was validated against follow‐up viral load data for a subset of n = 514 adolescents (all other medical records did not include recent viral load data). Viral load data that had been taken more than 1 year prior to the follow‐up interview were discarded from this analysis as outdated. After controlling for socio‐demographics, mode of HIV transmission, time on ART and HIV status awareness, retention in care was associated with and lower odds of virologic failure at follow‐up (AOR = 0.371; 95% CI: 0.224, 0.614).

### 3.3 Associations between discrimination multiplicity, internalized HIV stigma and retention in care

Cross‐tabulation results indicated graded, negative associations between discrimination multiplicity and retention in care. Among adolescents who reported no discrimination at baseline, 42.4% were retained in care; this proportion decreased to 31.1% among those experiencing one type of discrimination and to 20.3% among those experiencing multiple types of discrimination. Cross‐tabulation results also indicated a graded, positive relationship between discrimination multiplicity and internalized stigma: among adolescents experiencing no discrimination, 17.8% had internalized stigma and this increased to 32.5% and 54.5% among adolescents experiencing 1 or more types of discrimination respectively (Figure 1). Chi square tests indicated that these differences were significant at *p *< 0.001 level.

#### 3.3.1 Stage 3: Multiple regression results

Table 2 illustrates results of the logistic regression predicting retention in care. Baseline levels of all independent variables, including the hypothesized socio‐demographic and HIV‐related covariates, were used. Only discrimination in healthcare settings (OR = 0.539; *p* < 0.001) and internalized stigma (OR = 0.812; *p* < 0.005) were significantly associated with lower odds of retention in care. Neither discrimination due to the adolescent’s HIV status nor discrimination due to their family members’ HIV status were significantly associated with retention in care.

**Table 2:** Results of multiple logistic regression predicting long‐term retention in care (n = 995)

Independent variables	Odds ratio (OR)	95% Confidence Intervals (CIs)
*Socio‐demographic covariates*		
Age (>15 years)	1.02	0.74 to 1.40
Gender (female)	0.86	0.65 to 1.13
Location (rural)	0.86	0.63 to 1.17
*HIV‐related covariates*		
Mode of HIV transmission (horizontal)	0.79	0.52 to 1.19
Time on treatment (>1 year)	1.21	0.81 to 1.80
Adolescent knows his/her status	1.13	0.81 to 1.58
*Hypothesized discrimination predictors*		
Discrimination due to adolescent HIV status	1.23	0.70 to 2.13
Discrimination due to a family member’s HIV status	0.73	0.47 to 1.13
Discrimination in healthcare settings	0.54^a^	0.37 to 0.78
*Hypothesized mediator*		
Internalized stigma scale	0.81^a^	0.70 to 0.94

Model statistics: Chi‐square = 39.67; Nagelkerke R‐square = 0.06.

^a^ Denotes: *p* < 0.005.

### 3.4 Mediation analysis results

Results of the mediation analyses assessing direct and indirect effects for each type of discrimination on full retention in care are offered in Figure 2. There was no direct association between discrimination due to the adolescent’s HIV status and retention in care. However, both discrimination due to family HIV and discrimination in healthcare settings were directly associated with lower odds of retention in care. All three types of discrimination were indirectly associated with reduced retention in care, via internalized stigma.

## 4 DISCUSSION

This is the first quantitative study to examine multiple types of discrimination experienced by adolescents living with HIV, and their relationship to internalized stigma and long‐term retention in care. We found substantially lower rates of retention in care among adolescents than previous adult‐focused studies. 37.9% of adolescents in our study were retained in HIV care over the 2‐year period, whereas retention in care statistics for adults in this health district are 63.3–65.9% [35]. We found that three types of discrimination (in healthcare settings, due to the adolescents’ HIV status and due to the adolescents’ family members’ HIV status) all compromise retention in care via heightened internalized stigma. These findings add to the growing body of qualitative evidence, which has suggested that multi‐faceted discrimination that young people face results in profound feelings of shame that make it difficult to engage with life‐saving services [19, 36, 37, 38, 39, 40].

Future intervention research is urgently needed to improve outcomes for adolescents living with HIV globally. Specifically, more research is needed to understand how to best integrate clinic and community‐based responses to discrimination, internalized stigma and ART non‐adherence among adolescents living with HIV. Emerging evidence from Zimbabwe suggests that community adolescent treatment supporters help improve retention in care, adherence to ART and self‐worth among adolescents living with HIV [41]. A recent South African study found that provisions aligned with the sustainable development goals – tackling poverty, unemployment, health and violence – might help reduce mortality risk among adolescents living with HIV [4].

Addressing discrimination against adolescents living with HIV will require a substantial expansion of what are currently considered to be adolescent‐centred interventions. This will require a better understanding of the motivations behind harmful behaviours among health workers, community, peers and family members who all have an impact on young people’s lives. Robust research on how to effectively change those behaviours is essential. Impact evaluations of interventions that aim to change discriminatory behaviours often measure outcomes through self‐report from the people whose behaviours the researchers aim to change [42] but this is prone to social desirability bias. Incorporating young people’s perceptions of other people’s discriminatory behaviours would be helpful in improving validity of data. For example the Global Network of Young People Living with HIV have developed a scorecard for young people to assess the quality of health services and interactions with healthcare providers [43]. Moreover, the broader literature on bullying points to promising school‐based interventions focused on reducing maltreatment between peers [44]. But recent evidence suggests that stigma‐based bullying is worse for young people’s health outcomes than other types of bullying and that interventions require unique approaches [45]. More research on this is clearly needed.

All the associations between discrimination and compromised retention in care found in this study were mediated by internalized stigma. This finding suggests that routine screening for internalized stigma in healthcare settings might help healthcare providers identify young people in need of additional psychosocial support. Our findings further suggest that early intervention in the form of psychological support might help improve retention in care amongst young people who have experienced various forms of discrimination. Little is known about what might work to address internalized stigma among young people living with HIV but the broader, adult‐focused evidence base points to cognitive behavioural therapy, economic strengthening and community mobilization as promising approaches [46]. These should be adapted and tested for use among adolescents living with HIV.

This study has several limitations. First, although we aimed to measure multiple types of HIV‐related discrimination and internalized stigma, manifestations of stigma evolve across the continuum of care in ways that are difficult to capture in quantitative studies [47]. This paper focused on examining the effects of multiple types of discrimination and internalized stigma on retention in care. It did not examine all existing types of stigma; for example we did not examine anticipated stigma or the compounded stigma faced by young key populations. However, to our knowledge, this study includes the widest range of stigma types within existing quantitative studies on adolescent HIV in sub‐Saharan Africa. Second, following standard practice [42, 48], all of our discrimination variables were measured by adolescent self‐report. Therefore, it could be argued that these are measures of perceived rather than actual discrimination. Similarly, the retention in care outcome was based on adolescent self‐report, and therefore subject to the types of bias inherent in self‐reported data. However, the retention in care variable was validated against viral loads and we found a strong association between the two. Unfortunately, however, many clinic files were found to be missing or incomplete, so biomedical data were only available for a subset of adolescents. Third, our measurement of discrimination in healthcare settings relied on a single item in order to reduce participant burden. The question asked about being shouted at by your healthcare provider as this was a commonly reported form of discrimination in healthcare settings in the region [49, 50, 51, 52]. However, this measurement might have missed other forms of discrimination in healthcare settings.

Lastly, it should be noted that, while assumptions of causality are made for the mediation model, causality could not be confidently assumed or inferred from findings of the statistical analyses. The independent variables and mediator used for these analyses were based on cross‐sectional data collected at the same time point and the retention in care outcome was based on combined data from two time points. However, we built this model based on existing theory and empirical evidence. In the absence of large samples of adolescents living with HIV in Southern Africa with two or more follow‐up rounds, these findings provide valuable insight into how discrimination can compromise retention in care among youth.

## 5 CONCLUSIONS

This is the first quantitative study to shed light on the multiple types of discrimination and internalized stigma experienced by young people living with HIV, and the detrimental effect of these on engagement with government‐funded health services. Our findings add to the growing body of evidence that positions HIV‐related stigma as a serious threat to the United Nations Sustainable Development Goal on Health (SDG 3). Less than half (37.9%) of adolescents in this sample were retained in HIV care over the 2‐year period, whereas nearly a third (33.1%) experienced at least one form of discrimination in the past year. SDG 3 sets targets to end AIDS and achieve Universal Health Coverage including access to quality essential healthcare services for all by 2030. We found that different forms of discrimination heighten internalized stigma and compromise retention in care and contribute to virologic failure among adolescents living with HIV. More intervention research is urgently needed to better understand how to eliminate HIV‐related stigma and interrupt pathways of risk from discrimination to internalized stigma and non‐retention in care. Investment is also needed to design and test adolescent‐centred multi‐sectorial interventions so that young people living with HIV can live long and healthy lives in the era of efficacious ART.

## COMPETING INTERESTS

The authors have no competing interests.

## AUTHORS’ CONTRIBUTIONS

MP and MC drafted the manuscript, which was reviewed by all other authors. LC, ET and MP conceptualized the research project in which this study is embedded. LC, ET, MP and MC oversaw data collection. MP and MC led on the data analysis for this paper. MP, MM, MC, ET and LC contributed to the conceptualization of this paper and interpretation of findings. All authors have read and approved the final manuscript.
